# Spatiotemporal Up-Regulation of Mu Opioid Receptor 1 in Striatum of Mouse Model of Huntington’s Disease Differentially Affecting Caudal and Striosomal Regions

**DOI:** 10.3389/fnana.2020.608060

**Published:** 2020-12-10

**Authors:** Ryoma Morigaki, Jannifer H. Lee, Tomoko Yoshida, Christian Wüthrich, Dan Hu, Jill R. Crittenden, Alexander Friedman, Yasuo Kubota, Ann M. Graybiel

**Affiliations:** ^1^McGovern Institute for Brain Research, Massachusetts Institute of Technology, Cambridge, MA, United States; ^2^Department of Brain and Cognitive Sciences, Massachusetts Institute of Technology, Cambridge, MA, United States; ^3^Department of Advanced Brain Research, Institute of Biomedical Sciences, Graduate School of Medical Sciences, Tokushima University, Tokushima, Japan; ^4^Department of Neurosurgery, Institute of Biomedical Sciences, Graduate School of Medical Sciences, Tokushima University, Tokushima, Japan; ^5^Department of Neuroscience, Mayo Clinic, Jacksonville, FL, United States; ^6^Mayo Clinic Graduate School of Biomedical Sciences, Mayo Clinic, Jacksonville, FL, United States; ^7^Institute for Cancer Research, Massachusetts Institute of Technology, Cambridge, MA, United States

**Keywords:** mu opioid receptors, Huntington’s disease, neostriatum, movement disorders, mood disorders, animal models of human disorders, striosome

## Abstract

The striatum of humans and other mammals is divided into macroscopic compartments made up of a labyrinthine striosome compartment embedded in a much larger surrounding matrix compartment. Anatomical and snRNA-Seq studies of the Huntington’s disease (HD) postmortem striatum suggest a preferential decline of some striosomal markers, and mRNAs studies of HD model mice concur. Here, by immunohistochemical methods, we examined the distribution of the canonical striosomal marker, mu-opioid receptor 1 (MOR1), in the striatum of the Q175 knock-in mouse model of HD in a postnatal time series extending from 3 to 19 months. We demonstrate that, contrary to the loss of many markers for striosomes, there is a pronounced up-regulation of MOR1 in these Q175 knock-in mice. We show that in heterozygous Q175 knock-in model mice [~192 cytosine-adenine-guanine (CAG) repeats], this MOR1 up-regulation progressed with advancing age and disease progression, and was particularly remarkable at caudal levels of the striatum. Given the known importance of MOR1 in basal ganglia signaling, our findings, though in mice, should offer clues to the pathogenesis of psychiatric features, especially depression, reinforcement sensitivity, and involuntary movements in HD.

## Introduction

Huntington’s disease (HD) is an autosomal dominant neurodegenerative disease produced by a mutation of the gene *huntingtin* (*HTT*, Albin and Tagle, 1995). The expansion of a cytosine-adenine-guanine (CAG) repeat that encodes an expanded glutamine tract (PolyQ) in the mutant HTT protein elicits cellular dysfunctions including abnormal receptor signaling, mitochondrial stress, transcriptional dysregulation, protein trafficking and proteasome defects, and impairments in synaptic transmission, especially in basal ganglia circuits (Rub et al., 2015). Despite the expression of mutant *HTT* throughout the body, postmortem human pathology has shown the differential vulnerability of neuronal subpopulations in the forebrain, including the medium spiny neurons (MSNs, also called SPNs) of the striatum and some classes of striatal interneurons (Reiner et al., 1988, 2013; Albin et al., 1992). Also, several studies have documented differential neurodegeneration of the striosome and matrix compartments in the striatum, which could functionally affect the brain circuitry (Ferrante et al., 1985, 1987; Reiner et al., 1988, 2013; Goto et al., 1989; Albin et al., 1990; Morton et al., 1993; Hedreen and Folstein, 1995; Augood et al., 1996; Cicchetti et al., 2000; Tippett et al., 2007). In the anterior striatum, striosomes are preferentially innervated by cortical regions related to the limbic system, whereas innervation of the surrounding, much larger matrix compartment is biased toward inputs from sensorimotor and association regions (Ragsdale and Graybiel, 1981; Goldman-Rakic, 1982; Gerfen, 1984; Donoghue and Herkenham, 1986; Kincaid and Wilson, 1996). These findings have raised the possibility that dysfunction in the striosome compartment could contribute to the pathogenesis of psychiatric symptoms in HD and that striosome-related circuitry could be a candidate for treatment.

Pertinent to this possibility, the striatum has the highest levels of endogenous opioids and their receptors among brain regions (Mansour et al., 1995). A complex set of endogenous peptide precursors and their corresponding opioid receptor ligands are expressed throughout the striatum, but the mu-opioid receptor 1 (MOR1s) is notable in being highly enriched in striosomes, relative to the surrounding matrix. Ligands such as enkephalin, concentrated in the extrastriosomal matrix compartment, can bind to these receptors and have been shown to modulate both excitatory inputs from the cerebral cortex, thalamus and local inhibitory inputs to MSNs (Miura et al., 2008; Atwood et al., 2014; Banghart et al., 2015). Slice studies concur in showing that enkephalin signaling produces differential activation of striosomal over matrix MSNs *via* regulation of these inputs by both MORs and delta-opioid receptors (DORs).

At the protein level, MOR1s are enriched in direct pathway MSNs, where they have been shown to regulate opioid-driven dopamine release and self-administration (Cui et al., 2014). MOR1s are particularly enriched in direct pathway MSNs that make direct contact with dopamine-containing nigral neurons (Cui et al., 2014; Crittenden et al., 2016) to control their activity (McGregor et al., 2019). *Via* this pathway, the loss of striosomes in early-stage HD brains (Hedreen and Folstein, 1995) and in brains from HD individuals in which mood problems were early presentations of the disease (Tippett et al., 2007) would be expected to have a direct impact on the control of dopaminergic neurons and hence on the entire cortico-basal ganglia system.

The expression of the MOR1 in the striatum, in addition to being enriched in the striatum, exhibits a strong gradient of expression across the striatum, with its highest expression in the anterior and ventral parts of the caudoputamen and its lowest expression in the caudal part of the caudoputamen. These striatal gradient distributions of MOR1 are also likely important functionally for their potential clinical and therapeutic implications. Existing therapies to treat chorea in individuals with HD are aimed at reducing dopamine production but also carry a significant risk of depression (Huntington Study Group, 2006). The anterior striatal regions enriched in MOR1 are preferentially related to frontal, cingulate, and orbitofrontal cortex (and their respective mouse homologs, perhaps including parts of M2), whereas the more caudal regions of the striatum, poor in MOR1, are more related to sensory and motor cortical inputs. Thus, MOR1s are in a particularly strong position to regulate dopamine release related to limbic rather than sensorimotor functions, although other inputs not yet explored could have different distributions. How the striosomal gradients of opioid receptor signaling systems overlap with the differential loss of striosomes (Hedreen and Folstein, 1995) and opioids (Reiner et al., 1988) in early-stage HD patients is thus key to the development of therapies, not yet available, that can alleviate motor symptoms without worsening mood symptoms.

Here, to determine whether these functionally important receptors in the striatum are dysregulated in a mouse model of HD, we examined the caudoputamen of Q175 knock-in (Q175KI) mice (with 192.77 CAG repeats on average in this study), in which the number of CAG repeats has been engineered to be greatly expanded over those in wildtype (WT) mice. We analyzed the distribution of MOR1 protein by immunostaining in Q175KI mice and their sibling WTs across a wide range of ages, from 3 to 19 months old, corresponding roughly to 20–65 years old in the human. We found a striking age-related up-regulation of MOR1 immunoreactivity in striosomes across the entire caudoputamen, leading eventually to profound increases over normal at caudal levels that were nearly devoid of immunodetectable MOR1-positive striosomes in WTs. Thus, in this knock-in model of HD, there is a remarkable inversion of the normal gradient distribution of MOR1 as well as an intensification of its normal striosome-enriched compartmental distribution. These changes could have profound, progressive functional effects beginning early and then progressing during the course of the disorder.

## Materials and Methods

### Animals

All experimental protocols involving work with mice were approved by the Committee on Animal Care at the Massachusetts Institute of Technology (MIT). Four male and four female heterozygous Q175KI mice and matching numbers for WT littermates for each age (3, 6, 12, and 19 months postnatal) were obtained from Jackson Laboratory (Bar Harbor, ME, USA) or our colony at MIT.

### Immunizing Peptide Blocking Assay

The main series of experiments was performed with the rabbit monoclonal anti-MOR1 antibody raised against the 350–450 amino acid sequence of the human MOR1 C-terminal (ab134054, Abcam, MA, USA). To test for false-positive reactions, we confirmed the specificity of the immunostaining in blocking experiments using MOR peptide (ab239748, Abcam, MA, USA). The anti-MOR1 antibody (×500) was preincubated with MOR peptide (0.5 or 0.05 μg/ml) for 15 h in 3% bovine serum albumin (BSA) buffer before immunostaining. These preabsorbed solutions of primary antibody were used for immunostaining in striatal sections from WT mice as described below. The positive control was the section incubated with anti-MOR1 antibody (×500) in 3% BSA, and the negative control was the section incubated with 3% BSA.

### Immunohistochemistry

Mice were deeply anesthetized with an overdose of Euthasol (Virbac AH Inc., Westlake, TX, USA; pentobarbital sodium and phenytoin sodium) and were perfused with 0.9% saline, followed by 4% (wt/vol) paraformaldehyde in 0.1 M NaKPO_4_ buffer. Brains then were removed and post-fixed overnight with the same fixative at 4°C. They were stored in successive 10–30% sucrose solutions in 0.1 M NaKPO_4_ buffer for cryoprotection. Brains were cut into transverse 20 μm sections on a freezing microtome and were stored at 4°C in 0.1 M phosphate buffer saline (PBS) with 0.1% sodium azide until use. Immunohistochemical staining was performed on free-floating sections. After blocking endogenous peroxidase activity with H_2_O_2_, the sections were incubated in PBS containing 3% BSA for 60 min. Sections were then incubated in the ab134054 (Abcam, MA, USA) rabbit monoclonal anti-MOR1 antibody (1:200) for 15 h at 4°C. The sections were then treated with anti-rabbit secondary antibody conjugated to polymers of horseradish peroxidase (Invitrogen, Carlsberg, CA, USA) and bound peroxidase was detected by incubating the sections with a solution containing 3,3′-diaminobenzidine (DAB) with nickel (Vector, Burlingame, CA, USA) and 0.01% H_2_O_2_ for 10 min. For fluorescence immunostaining, sections were incubated in rabbit monoclonal anti-MOR1 antibody (1:10,000) for 15 h at 4°C. The bound primary antibodies were detected by the same secondary antibody and the tyramide signal-amplification system with Cyanine 3 (Perkin Elmer, Shelton, CT, USA).

Rodent MOR1 has several alternative splicing variants (Pasternak, 2018). To increase the informativeness of the immunostaining experiments, we also performed experiments with a rabbit polyclonal anti-MOR1 antibody raised against mouse N-terminal 1–38 amino acid sequence (×200; MOR-Rb-Af240, Frontier Institute Company, Limited., Hokkaido, Japan; Kasai et al., 2011). The DAB processing was performed with nickel enhancement.

### Digital Images and Analysis

**Figure 1:**
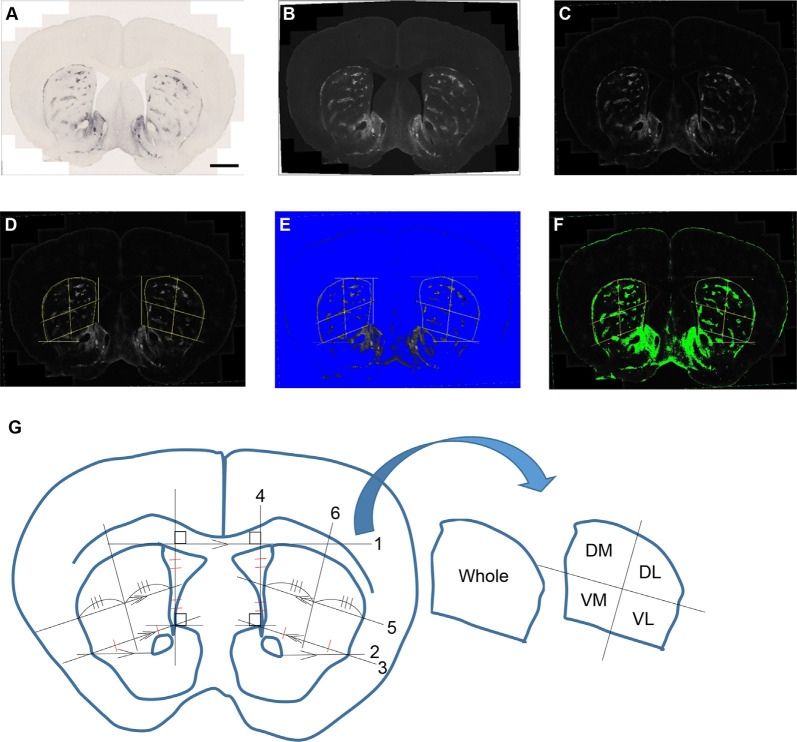
An example of processing a raw image. The obtained raw image **(A)** was converted into a 16-bit grayscale and was inverted **(B)**. The background signals were subtracted using the “Rolling Ball” command (radius = 500 pixels) of Fiji software. Small noise was removed by the “Median Filter” command (radius = 5 pixels; **C**). Each area was manually defined and divided into four areas for the bilateral caudoputamen **(D,G)**. The threshold was defined in the image from each area using the Li method for the demarcation of striosome **(E)** and matrix **(F)** compartments. Analyses of the percentage area of striosomes and densitometric data of both compartments were performed for each area. The manual demarcation of the caudoputamen was performed as described below **(G)**: (1) Draw the tangent line (line 1) between right and left caudoputamen. (2) Draw the parallel line (line 2) to line 1 from the lateral tip of the anterior commissure. (3) Draw the line (line 3) from the upper tip of the nucleus accumbens shell to the cross point of line 2 and the lateral margin of the caudoputamen. (4) Draw the line (line 4) vertically to line 1 from the upper tip of the nucleus accumbens shell. (5) Draw the parallel line (line 5) to 3 from the midpoint of 4. (6) Divide line 4 and the striatal portion of lines 3 and 5 evenly. (7) Draw the line (line 6) going through the midpoints of lines 3 and 5 as defined in (5). (8) Repeat (2–7) on the other side. (9) Outline manually whole, dorsomedial (DM), dorsolateral (DL), ventromedial (VM), and ventrolateral (VL) caudoputamen and save it to the ROI manager.

Microscopic images were captured with a TissueFAXS whole-slide scanning system equipped with a Zeiss Axio Imager Z2 fluorescence microscope and TissueFAXS image acquisition software. The acquired images were processed and analyzed with Fiji software (Schindelin et al., 2012). MOR1 labeling detected by rabbit monoclonal anti-MOR1 antibody was measured in the striosomal and matrix subfields in the striatum for each mouse (*n* = 8, each with both hemispheres, yielding 16 caudoputamen samples) across each age (3, 6, 12, and 19 months). Two specific brain regions at rostral and caudal levels of the caudoputamen were selected for each WT and Q175KI mouse. Coronal sections at +1.10 and −0.34 mm to the Bregma (Franklin and Paxinos, 2008) were evaluated histologically. We arranged 24 serial sections (20 μm) at, respectively, rostral and caudal levels in a 96 well plate for each mouse and selected the brain region of these coordinates as nearly identical as possible. MOR1-positive area and intensity were defined using Fiji software (Figure 1). Images were converted into 16-bit gray levels and were inverted (Figures 1A,B). The background signals were subtracted using the “Rolling Ball” command (radius = 500 pixels). Small noise was removed by use of the “Median Filter” command (radius = 5 pixels; Figure 1C). Four regions in the caudoputamen (dorsomedial, dorsolateral, ventromedial, and ventrolateral) were manually defined (Figure 1D). This part of the analysis was based on the subjective definition and was performed blinded to genotype. Then the MOR1-immunopositive regions were objectively defined. For the images, thresholds were set using the Li method, which enabled the demarcating of striosome and matrix compartments (Li and Tam, 1998), for each region (i.e., whole, dorsomedial, dorsolateral, ventromedial, and ventrolateral caudoputamen) independently (Figures 1E,F).

Analyses of area and densitometry were performed, respectively, for the striosome and matrix compartments, excluding fiber bundles. For densitometric analyses, 50 μm squares were set on the corpus callosum and anterior commissure for each caudoputamen. The intensity of these two squares was averaged, and the obtained value was truncated after the decimal point. The integral value was used as a threshold for excluding non-neuronal fiber.

### Statistical Analysis

All quantitative data were expressed as means ± SEM values. Kruskal–Wallis tests followed by pairwise Mann–Whitney *U*-tests with Bonferroni corrections were used. *P*-values less than 0.05 were adjusted by Bonferroni methods and were considered as statistically significant.

## Results

**Figure 2:**
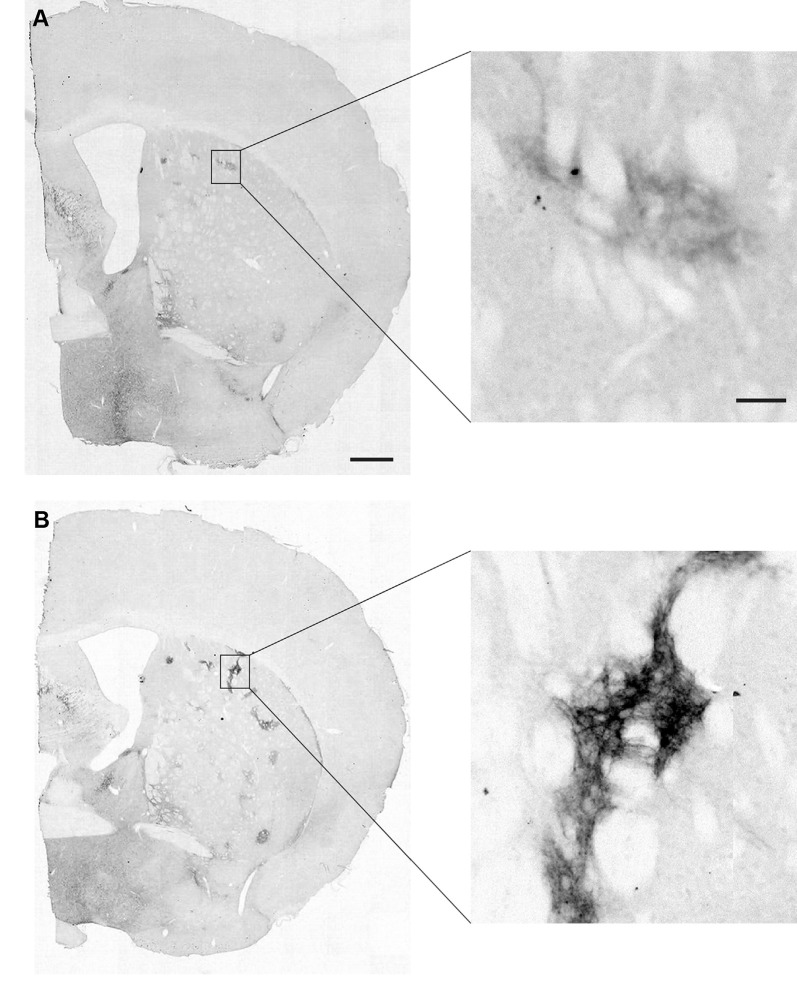
Mu-opioid receptor 1 (MOR1) labeling in the caudal caudoputamen of wildtype (WT) and Q175KI mice at 12 months of age. Almost no MOR1-immunopositive striosomes were visible in WT mice **(A)**, whereas strong MOR1 immuno-labeling was detected in striosomes of the Q175KI mouse **(B)**. Scale bars = 500 μm (left) and 50 μm (right).

**Figure 3:**
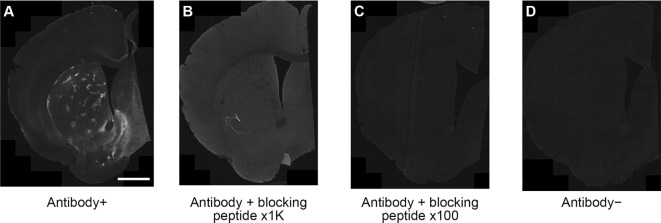
An immunizing peptide blocking assay. In comparison with positive control staining with anti-MOR1 antibody **(A)**, the labeling of MOR1 was blocked when the primary antibody was preabsorbed with 0.05 μg/ml **(B)** and 0.5 μg/ml **(C)** MOR1 peptide antigen. Note that the pre-absorption with high-dose peptide antigen **(C)** completely blocked MOR1 labeling equivalent to the level of negative control staining **(D)**. Raw images were inverted, subtracted from their background, and filtered by using Fiji software. Scale bar = 1 mm.

Striatal MOR1 immunostaining was performed at maturity in the heterozygous Q175KI mice and their WT control mice at the age of 12 months (Figure 2). MOR1-positive striosomes were hardly visible at this mid-caudal level, except for the chain of relatively weak striosomes near the dorsal border of the caudoputamen (Figure 2A). By contrast, in the sibling Q175KI mouse, the MOR1-positive striosomes along this dorsal rim were intensely immunostained, and MOR1-positive striosomes were also visible at deeper levels within the striatum, gradually fading ventrally (Figure 2B). This strikingly higher intensity of striosomal staining in the Q175 mutant relative to its control sibling was characteristic of all Q175 mutant-WT sibling pairs. To test for the selectivity of this MOR1 immunoreactivity, we used the antigen pre-absorption procedure. We found a dose-dependent reduction of immunostaining with pretreatment with recombinant human MOR1 protein (Figures 3A–D). MOR1 immunostaining levels with pre-absorption were equivalent to those measured in negative controls when the anti-MOR1 antibody was preincubated with MOR1 protein at the dilution rate of 0.5 μg/ml (Figures 3C,D).

### MOR1 Expression in the Rostral Caudoputamen Across the 3–19 Month Age Series for WT and Q175KI Mice

We asked whether this remarkable up-regulation of striosome immunostaining occurred only in adulthood, or instead, was the end-product of a gradual process occurring throughout development. We first examined the possibility that the apparent increase in MOR1-positive striosomes in the Q175KI HD model was due to a change in their size with disease progression, mindful that MOR1 itself has proven to be a reliable marker for striosomes in the caudoputamen of rodents. At the anterior levels, the areal extent of MOR1-positive zones has been considered as equivalent to those of striosomes. We, therefore, quantified the MOR1-immunostained areas at four different ages, comparing values for WT and Q175KI mice at +1.1 mm along the anterior-posterior axis relative to the Bregma (Franklin and Paxinos, 2008). For this sake, the anterior caudoputamen was divided into four regions (Figure 1G).

**Figure 4:**
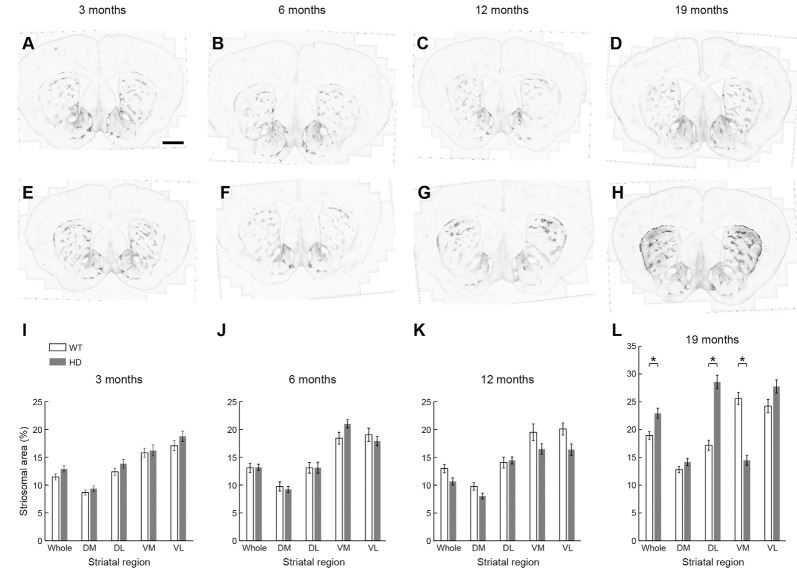
Histological evaluation of the MOR1 labeling across four different ages (3, 6, 12, and 19 months) in the whole, dorsomedial (DM), dorsolateral (DL), ventromedial (VM), and ventrolateral (VL) regions of the rostral caudoputamen of WT and Q175KI mice. The background signals were subtracted using the “Rolling Ball” command (radius = 500 pixels) of Fiji software. At 3, 6, and 12 months of age (WT: **A–C**, Q175KI: **E–G**), there was no difference between the size of the MOR1-positive striosome zones between WT and Q175KI mice **(I–K)**. By 19 months of age, the total MOR1-immunoreactive area increased in Q175KI mice **(H)** relative to WT mice **(D)** when either the whole (*p* = 0.001) or the dorsolateral (*p* < 0.001) caudoputamen was examined **(L)**. However, the total MOR1-immunoreactive area decreased in the ventromedial caudoputamen (*p* < 0.001; **D,H,L**). Asterisks indicate significant differences (*p* < 0.005 after Bonferroni correction). Processed images were inverted. Scale bar = 1 mm.

We found no difference in the size of the MOR1-positive striosomal zones between the WT (*n* = 8, 16 caudoputamen) and Q175KI (*n* = 8, 16 caudoputamen) mice at any of the 3, 6, and 12-month ages sampled (Figures 4A–C,E–G,I–K). By 19 months of age, however, the total MOR1-immunopositive areas per total regions of the same sections were significantly increased relative to WT values for either the entire caudoputamen (*p* = 0.001) or its dorsolateral quadrant (*p* < 0.001; Figures 4D,H,L). By contrast, the total MOR1-immunoreactive area in the ventromedial caudoputamen of Q175KI mice was markedly decreased compared to WT (*p* < 0.001; Figures 4D,H,L).

**Figure 5:**
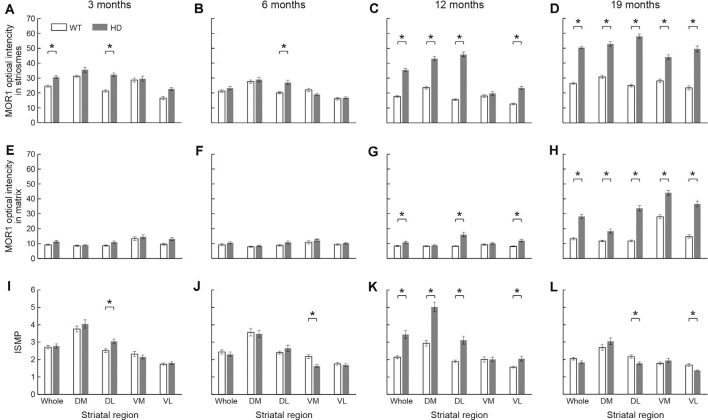
The densitometric analysis across four different ages in the rostral caudoputamen of WT and Q175KI mice. The intensity of the MOR1-immunopositive (striosomal) zones increased in the whole (*p* < 0.001) and dorsolateral (*p* < 0.001) caudoputamen at 3 months of age **(A)**, in the dorsolateral caudoputamen (*p* = 0.001) at 6 months of age **(B)**, in all areas (*p* < 0.001) except the ventromedial caudoputamen at 12 months of age **(C)**, and in all areas (*p* < 0.001) at 19 months of age **(D)** in Q175KI mice, relative to WT mice. The intensity of the MOR1-immunonegative (matrix) zones was not different between WT and Q175KI mice at 3 and 6 months of age **(E,F)**. The intensity of the MOR1-immunonegative zones increased in the whole (*p* = 0.002), dorsolateral (*p* < 0.001) and ventrolateral (*p* < 0.001) caudoputamen at 12 months of age **(G)**, and in all segments at 19 months of age (*p* < 0.001; **H**) in Q175KI mice, relative to WT mice. The index of striosome-to-matrix predominance (ISMP) increased in the dorsolateral caudoputamen (*p* = 0.004) at 3 months of age **(I)**, but it decreased in the ventromedial caudoputamen (*p* < 0.001) at 6 months of age **(J)** in Q175KI mice relative to WT mice. The ISMP strikingly increased in the whole (*p* < 0.001), dorsomedial (*p* < 0.001), dorsolateral (*p* < 0.001), and ventrolateral (*p* = 0.002) caudoputamen at 12 months of age **(K)**. The ISMP was normalized in the whole, dorsomedial and ventromedial caudoputamen (*p* > 0.005), but it was still higher in the dorsolateral (*p* = 0.004) and ventrolateral (*p* = 0.001) caudoputamen at 19 months of age **(L)** in Q175KI mice than in WT mice. Asterisks indicate significant differences (*p* < 0.005 after Bonferroni correction).

Next, we tested whether the intensity of MOR1 immunoreactivity changed with disease progression in the Q175KI mice. We made densitometric measurements of the MOR1-immunoreactive zones for the rostral caudoputamen of WT (*n* = 8, both hemispheres) and Q175KI (*n* = 8, both hemispheres) mice (Figures 4A–H, 5). At these anterior levels, an up-regulation of MOR1 immunostaining was apparent in the older mice. We examined MOR1 fluorescence immunostaining using the same antibody (Supplementary Figure 1). Also, DAB immunostaining with rabbit polyclonal anti-MOR1 antibody raised against the mouse N-terminal sequence was performed (Supplementary Figure 2). The immunostaining patterns with the two antibodies were similar, with MOR1 staining of striosomes becoming weak caudally, and with this immunostaining being strongly up-regulated in the older Q175KI mice. These visual impressions were confirmed by densitometric analysis in the main series of experiments performed with the rabbit monoclonal anti-MOR1 antibody raised against the human MOR1 C-terminal (ab134054, Abcam, MA, USA).

In 3-month-old mice, the intensity of MOR1 immunostaining marking striosomes was already up-regulated in the caudoputamen and especially in the dorsolateral caudoputamen of the Q175KI mice relative to their WT controls (*p* < 0.001; Figures 4A,E, 5A). By contrast, there was no genotype difference for the intensity of MOR1 immunostaining in the matrix at the 3-month-old timepoint (Figures 4A,E, 5E). The relative ratio of striosome and matrix intensities, measured as an index of striosome-to-matrix predominance (ISMP), was increased significantly only in dorsolateral caudoputamen at these mid-anterior levels in the 3-month-old Q175KI mice (*p* < 0.01; Figures 4A,E, 5I). We looked for, but did not find, a detectable difference in the MOR1 immunostaining between Q175KI mice and WT siblings at 2 months (data not shown).

At the age of 6 months, the MOR1 immunoreactivity of striosomes was further increased in the dorsolateral caudoputamen in Q175KI mice in comparison with WT mice (*p* < 0.01), whereas, again, there was no detectable difference in the intensity of matrix MOR1 immunoreactivity (Figures 4B,F, 5B). Thus, the MOR1 immunoreactivity of striosomes began to increase earlier relative to that of the matrix (Figures 4B,F, 5F). Again, the ISMP was decreased in the ventromedial caudoputamen in Q175KI relative to WT mice (*p* < 0.001; Figures 4B,F, 5J).

By 12 months of age, the intensity of MOR1 immunostaining in striosomes was strikingly increased across the entire area of the caudoputamen at the levels sampled, including the dorsomedial, dorsolateral and ventrolateral sectors, in Q175KI compared with WT mice (*p* < 0.001; Figures 4C,G, 5C). The extent of the up-regulation of MOR1 immunoreactivity in striosomes in comparison to that of the matrix compartment was prominent by this age (Figures 4C,G, 5G). Nevertheless, the intensity of MOR1 immunoreactivity in the matrix was also up-regulated as calculated for the full area of the caudoputamen examined (*p* < 0.01), as well as in individual sectors for the dorsolateral (*p* < 0.001) and ventromedial (*p* < 0.001) caudoputamen. The ISMP was significantly increased in whole, dorsomedial, dorsolateral (*p* < 0.001) and ventrolateral (*p* < 0.01) caudoputamen (Figures 4C,G, 5K). Again, the MOR1 immunoreactivity both in the ventromedial striosomes and in the matrix adjacent to the nucleus accumbens core was not detectably different from the levels in the WT controls (Figures 5C,G).

At the latest stage that we examined, 19 months, the intensity of MOR1 immunostaining of the striosomes was yet further increased across all caudoputamen regions in the Q175KI mice in comparison with that of WT mice (*p* < 0.001; Figures 4D,H, 5D). The immunostaining in the matrix was also markedly increased throughout the caudoputamen (*p* < 0.001), and in individual sectors including the dorsomedial, dorsolateral and ventrolateral sectors (*p* < 0.001) and even the ventromedial sector (*p* < 0.001; Figures 4D,H, 5H). The simultaneous up-regulation of MOR1 immunoreactivity in the matrix compartment at 19 months relative to 12 months of age reduced the ISMP in the Q175KI mice (Figures 5G,H,L). The ISMP returned to normal in the full caudoputamen at the levels sampled, in the dorsomedial and ventromedial caudoputamen, and even reached below the levels of WTs in the dorsolateral (*p* < 0.01) and ventrolateral (*p* < 0.01) caudoputamen (Figure 5L). Thus, by these late stages of the disorder, the MOR1 in the matrix was as affected, or even more so, as MOR1 in striosomes. Altogether, these findings for the anterior caudoputamen suggest that the increased intensity of striosomal MOR1 immunostaining began to be detectable by 3 months of age in the Q175KI model of HD and was saturated by 12 months of age, whereas the MOR1 intensity in the matrix began to increase noticeably only after 12 months of age. Notably, the ventromedial caudoputamen exhibited contradictory changes, with a decreased ISMP at 6 months of age and decreased striosomal areas at 19 months of age (Figures 4L, 5J). These findings might indicate differential mechanisms and consequences for the MOR1 changes occurring in this region, situated close to the nucleus accumbens core.

At these anterior levels, and excepting the ventromedial sector, there was, relative to WT levels, strong up-regulation in the intensity of MOR1 immunostaining by 12 and 19 months of age that preceded the increase in immunostaining of the matrix, detectable at 19 months of age in Q175KI mice compared to WT mice. Strikingly, the ISMP in the ventromedial sector just adjacent to the nucleus accumbens core, which is responsible for the evaluation of reward and initialization of reward-related motor action (Scofield et al., 2016), increased at 12 months of age and was normalized at 19 months of age (Figures 5K,L). Together, these results suggest that there would be an imbalance in MOR1 signaling in striosomes over matrix early in the course of the disease that, by late stages becomes more wide-spread across the compartments.

### Striking Up-Regulation of MOR1 Expression in Striosomes in the Caudal Caudoputamen Q175KI Mice Across Age Relative to Levels in WT Siblings

**Figure 6:**
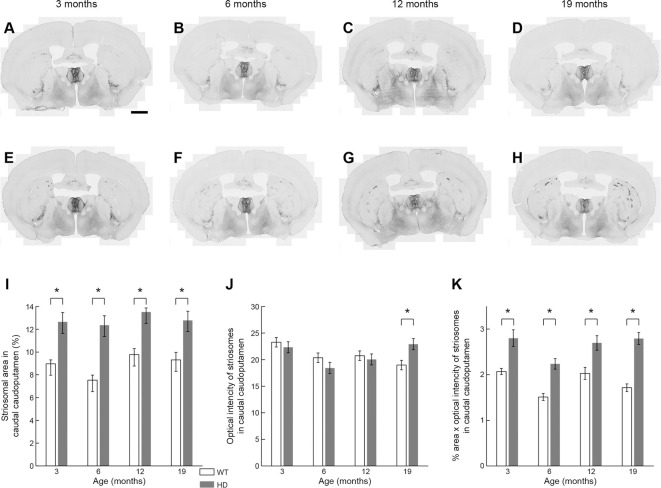
Histological evaluation of the MOR1 labeling across four different ages in the caudal caudoputamen of WT and Q175KI mice. The background signals were subtracted using the “Rolling Ball” command (radius = 500 pixels) of Fiji software. At all ages (3, 6, 12, and 19 months), the MOR1-immunopositive area increased in Q175KI **(E–H)** relative to WT **(A–D)** mice **(I)**. The mean intensity of the MOR1-immunopositive area was increased only at 19 months (*p* = 0.003; **J**). The whole intensity of MOR1-immunopositive area (intensity × %area) increased at all ages (*p* < 0.001 at 6 and 19 months, *p* = 0.001 at 3 months, and *p* = 0.006 at 12 months of age; **K**). Asterisks indicate significant differences (*p* < 0.005 after Bonferroni correction). Processed images were inverted. Scale bar = 1 mm.

It was obvious at a glance that MOR1-positive striosomes were visible in the most caudal striatal sections from Q175KI mice, but hardly so in the WT controls (Figure 2). This difference was clear across all ages (Figure 6). MOR1 immunostaining was scarcely detectable or not detectable in the caudal caudoputamen of WT mice. In sharp contrast, in the Q175KI mice, MOR1-positive striosomes were prominent throughout these caudal regions (Figures 2, 6A–H). The full caudoputamen measurements of MOR1 immunostaining were significantly increased, relative to the WT levels, in the Q175KI mice already at 3 months of age (*p* < 0.001), and the MOR1-positive striosome enrichment was maintained across all ages (*p* < 0.001 at 3, 6, and 12 months, and *p* = 0.003 at 19 months; Figure 6I). The mean intensity of the MOR1-immunopositive area was increased only at 19 months (*p* < 0.01), and the total intensity of the MOR1-immunopositive area (intensity × %area) increased at all ages (*p* < 0.001 at 6 and 19 months, *p* < 0.01 at 3 months, and *p* < 0.01 at 12 months of age; Figures 6J,K).

## Discussion

The Q175KI mouse was derived from a spontaneous expansion of germline CAG trinucleotide from the CAG140 KI mouse model, which was constructed by replacing the endogenous mouse huntingtin (*Hdh*) exon 1 with a chimeric human/mouse *HTT* exon 1 carrying the 140 CAG repeat region and the human polyproline region (Menalled et al., 2012). Q175KI mice exhibit a slower progression of the disease than do many other mouse models of HD (Menalled et al., 2012; Smith et al., 2014). These dynamics render Q175KI mice well suited for tracking the progressive functional and neurodegenerative changes that emerge over time (Smith et al., 2014). Our findings demonstrate that the up-regulation of MOR1 immunostaining in the striosomes of the Q175KI mice is first detectable in the caudoputamen at 3 months of age, followed by an increase in the matrix at 12 months of age. The detection of up-regulation already at 3 months suggests that this change is one of the early signs so far identified. Carty et al. (2015) have found that EM48, a mutant HTT protein maker, is visible, but not aggregated, in the nucleus of striatal cells at 3 months of age and is aggregated by 4 months of age in Q175KI mice (Langfelder et al., 2016). Only a minority of RNA and protein changes have been detected at 2 months of age, but by 6 months, many changes have been shown (Langfelder et al., 2016). Thus, the up-regulation of MOR1 in the Q175KI striatum is an early change, suggesting that it might not be, at least initially, solely compensatory. Also, this change is a histological confirmation of instances of up-regulation, as opposed to down-regulation, of neuronal proteins seen in proteomic and PET studies in HD and in the Q175KI HD model (Ferrante et al., 1985, 1987; Reiner et al., 1988, 2013; Goto et al., 1989; Albin et al., 1990, 1992; Morton et al., 1993; Hedreen and Folstein, 1995; Augood et al., 1996; Cicchetti et al., 2000; Tippett et al., 2007; Smith et al., 2014; Langfelder et al., 2016; Wilson et al., 2017; Padovan-Neto et al., 2019). Given that MOR1 mRNA in Q175KI mice was not up-regulated in the striatum at the age of 2, 6, and 10 months (Langfelder et al., 2016), as judged by immunohistochemistry, MOR1 up-regulation in our study may be thought to occur in posttranscriptional or posttranslational manner, for example, due to dysregulation in alternative splicing, the consequence of protein-protein interaction, or compensation to the changes in the expression levels in other proteins. Again, the up-regulation of MOR1 that begins as early as 3 months of age in the striosome compartment before the aggregation of mutant Htt suggests that this change occurs due to the transcriptional dysregulation. However, we should be careful about the results of mRNA studies because they are largely affected by the methods of sampling and have difficulty in showing the difference between very small structures such as striosomes and matrix in the caudoputamen. The technique to measure the mRNA of these small structures based on snRNS-seq might resolve these problems in the future (Gokce et al., 2016; Saunders et al., 2018).

Q175KI mice exhibit motor deficits at ~6 months of age (Smith et al., 2014). By 12 months, Q175KI mice manifest motor as well as “cognitive” deficits paralleled by detectable postmortem striatal atrophy, cortical thinning, degeneration of MSNs, dense mutant HTT inclusion formation, decreased striatal dopamine levels, and loss of striatal brain-derived neurotrophic factor (Smith et al., 2014). By this time, the changes in MOR1 have become obvious at both anterior and posterior levels.

MOR1 immunostaining in striosomes, relative to undetectable increases in the matrix compartment in the same brains, was a common feature of the progressive up-regulation of MOR1 immunostaining from 3 months up to late ages, when MOR1 immunostaining was also strongly elevated in the matrix. Striosomes in the anterior caudoputamen receive differentially prominent inputs from limbic structures (Crittenden and Graybiel, 2011), but the inputs to caudal striosomes, not fully mapped, apparently include afferents from non-limbic cortical regions. The gene expression profiles of the caudal striosomes are also different from those of anterior striosomes (Tajima and Fukuda, 2013). Great caution must therefore be taken in interpreting any striosomal changes without knowledge of their regional location and the different gradients of expression that they exhibit; and similar caution must be used in assuming that such functional changes are “limbic” related, although for the anterior striatum this possibility is likely. It also should be noted that at the anterior striatal levels we examined, the earliest MOR1 up-regulation was mainly in the dorsolateral part of the caudoputamen. Moreover, we found that the MOR1 up-regulation eventually was visible in most parts of the caudoputamen but was not evident in the ventromedial caudoputamen, next to and likely blending into the core of the nucleus accumbens, until late stages. If there is a relationship between the MOR1 changes and behavioral signs progressively emerging, then manipulation of the opioid system might selectively decrease symptoms or even slow the process of neurodegeneration in the caudoputamen. These differences could imply selective behavioral deficits that could become biomarkers for disease progression in HD (Nandhu et al., 2010).

Involvement of the striosome compartment in the pathogenesis of HD has been implicated so far by anatomical measurements of postmortem specimens (Morigaki and Goto, 2017). In the early stage of the disease, a preferential loss of striosomal neurons that gradually spreads to the matrix neurons has been reported in such postmortem HD striatal specimens (Goto et al., 1989; Morton et al., 1993; Hedreen and Folstein, 1995; Augood et al., 1996; Tippett et al., 2007). A potential relationship between pronounced striosomal neuronal loss and mood disturbance was suggested in a small number of postmortem brains from patients with HD (Tippett et al., 2007). Preferential loss of MSNs in striosomes relative to matrix has also been reported for the postmortem striatum in X-linked dystonia-parkinsonism (Goto et al., 2005, 2013), and it has been hypothesized that this decrease could be an important factor in the development of abnormal involuntary movements (Graybiel et al., 2000; Crittenden and Graybiel, 2011). More studies of these putative patterns are needed.

Specific molecules and neurotransmitters have been shown to change differentially in the striosomes and matrix of postmortem HD patients. Enkephalin, substance P, and nicotinamide adenine dinucleotide phosphate diaphorase are reduced greatly in the striosome compartment in the rostral caudoputamen of postmortem HD specimen (Ferrante et al., 1986; Morton et al., 1993; Tippett et al., 2007). Enkephalin is a primary endogenous ligand for MORs and DORs (Banghart et al., 2015). Therefore, it is reasonable for cell-surface MOR1 to be increased as a result of a reduction in ligand-binding-mediated internalization and proteolytic degradation. After dopamine depletion, there is a pronounced up-regulation of enkephalin in the indirect pathway MSNs that is thought to be responsible for the compensatory down-regulation of both MORs and DORs in their target cells (Hollt, 1986; Koizumi et al., 2013). Rather than being constitutively low as in Parkinson’s disease, dopamine levels appear to fluctuate in HD (Bird et al., 1980; Garrett and Soares-da-Silva, 1992; Andre et al., 2010; Schwab et al., 2015), and dopamine depletors can be therapeutic for chorea but frequently contribute to symptoms of depression. The two therapeutic drugs so far FDA-approved are tetrabenazine and deutetrabenazine that deplete presynaptic dopamine by blocking vesicular monoamine transporter type 2 (Claassen et al., 2019). Thus our work, albeit in a mouse model of HD, suggests that, as in Parkinson’s disease, changes in MOR1 expression levels could occur as a consequence of dopamine dysregulation. Besides, MOR1s are expressed not only in striatal projection neurons but also in axon terminals in the striatum originating in the neocortex and the thalamus (Birdsong et al., 2019; Muñoz et al., 2020). Therefore, MOR1 up-regulation might occur in these presynaptic terminals to suppress excessive excitation of these presynaptic neurons. Further work with snRNA-seq and other methods could help to resolve these issues.

Striosomes in immunostained sections from the human, monkey, and cat striatum typically have relatively enkephalin-poor centers with enkephalin-rich rims, surrounded by the enkephalin-positive matrix, with the levels sensitive to immunostaining methods (Graybiel et al., 1981; Haber and Elde, 1982; Gerfen, 1984; Graybiel and Chesselet, 1984; Inagaki and Parent, 1985; Groves et al., 1988; Martin et al., 1991; Holt et al., 1997; Prensa et al., 1999). In mice, relatively low expression levels of enkephalin in striosomes compared to matrix have been noted (Koshimizu et al., 2008; Tajima and Fukuda, 2013; Banghart et al., 2015). Enkephalin-induced MOR1 modulation in the striosome compartment has been reported to have only a minor role in suppressing neuronal activities (Banghart et al., 2015). Enkephalin in the striatum has been shown to have neurotransmitter-like properties in studies showing potassium-induced release (Henderson et al., 1978), and enkephalin is thought to modulate both direct and indirect pathway neurons *via* paracrine transmission (McCollum et al., 2012). MOR1s in the striosome compartment are enriched in direct pathway MSNs (Guttenberg et al., 1996; Cui et al., 2014), which could directly modulate dopamine-containing nigral neurons (Crittenden et al., 2016; McGregor et al., 2019). The amount of dopamine release in the matrix compartment, while affecting both compartments, might have a major effect on the striosomal neurons projecting to the dopamine neurons, thus producing decreases or rebound increases in dopamine neuron activity (Crittenden et al., 2016; McGregor et al., 2019).

In conclusion, this immunohistochemical study demonstrates that there are striking, and notably selective, changes in the expression of MOR1 in the striosome compartment throughout the caudoputamen in Q175KI mice, and a remarkable age-dependent MOR1 up-regulation, most notably in the caudal caudoputamen. Our findings could provide a new insight related to the genesis of psychiatric symptoms including depression, and in dopamine fluctuations, reward and cost sensitivity, and movement disabilities in HD.

## Data Availability Statement

The raw data supporting the conclusions of this article will be made available by the authors, without undue reservation.

## Ethics Statement

The animal study was reviewed and approved by Committee on Animal Care at the Massachusetts Institute of Technology.

## Author Contributions

RM, JL, JC, and AG designed the study. RM, JL, CW, DH, AF, and TY performed the immunohistochemical staining. RM, JL, and JC performed data analysis. RM, JL, TY, JC, YK, and AG prepared the figures and drafted the manuscript. All authors contributed to the article and approved the submitted version.

## Conflict of Interest

The authors declare that the research was conducted in the absence of any commercial or financial relationships that could be construed as a potential conflict of interest.

## Abbreviations

HD: Huntington’s disease
MOR1: mu opioid receptor 1
DOR: delta opioid receptor
CAG: cytosine-adenine-guanine
HTT: huntingtin
MSN or SPN: medium spiny neuron
WT: wild type
Q175KI: Q175 knock-in
MIT: Massachusetts Institute of Technology
BSA: bovine serum albumin
PBS: phosphate buffer saline
DAB: 3,3^′^-diaminobenzidine
ISMP: index of striosome-to-matrix predominance.
