# Supplementary material for: Spatiotemporal Up-Regulation of Mu Opioid Receptor 1 in Striatum of Mouse Model of Huntington’s Disease Differentially Affecting Caudal and Striosomal Regions
Source: Front Neuroanat. 2020 Dec 10;14:608060. doi: 10.3389/fnana.2020.608060 (PMC7758501; doi:10.3389/fnana.2020.608060)
Supplement: Supplementary file 1 [file Image_1.pdf]

Supplementary Material

1. Supplementary Figures

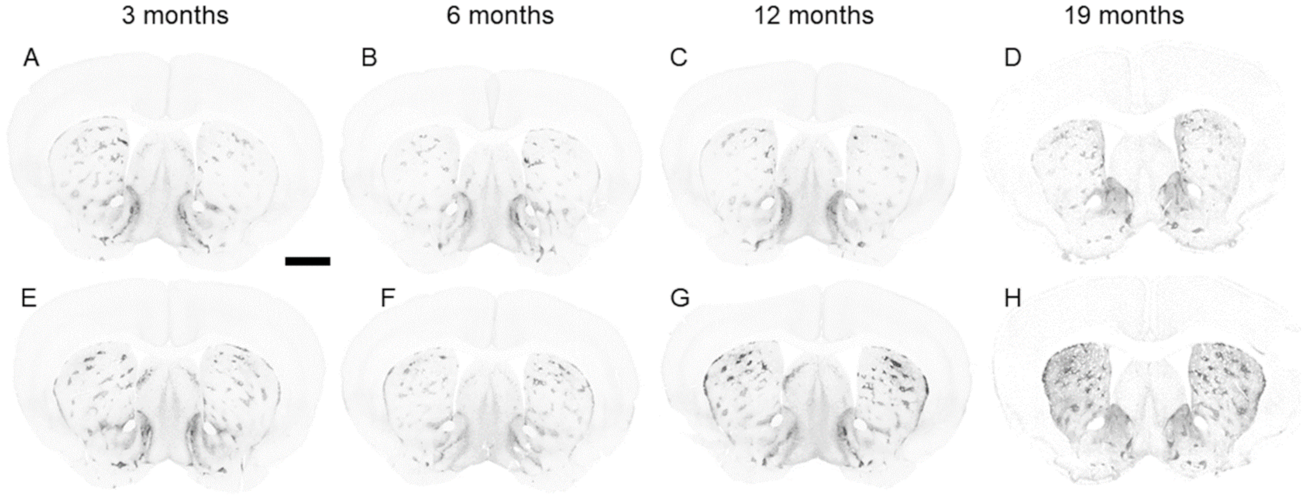

Supplementary Figure 1. MOR1 labeling in the rostral caudoputamen of WT (A-D) and Q175KI (E-H) mice across four different ages (3, 6, 12, and 19 months) using fluorescent TSA method. Images were converted into 8-bit gray scale and inverted. The background signals were subtracted using the "Rolling Ball" command (radius = 500 pixels) of Fiji software. Scale bar = 1 mm.

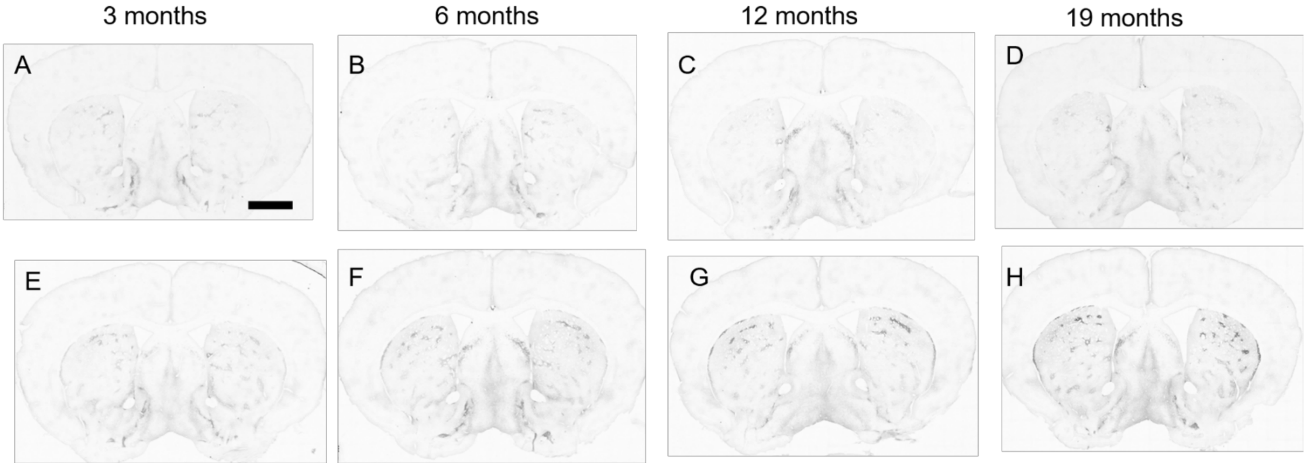

Supplementary Figure 2. MOR1 labeling in the rostral caudoputamen of WT (A-D) and Q175KI (E-H) mice across four different ages (3, 6, 12, and 19 months) using anti-MOR1 antibody raised against mouse N-terminal sequence. 3,3'-diaminobenzidine (DAB) with nickel staining was used. Images were converted into 16-bit gray scale. The background signals were subtracted using the "Rolling Ball" command (radius = 500 pixels) of Fiji software. Scale bar = 1 mm.
